# Metabolite Profiling Under Dietary Myo-Inositol Supplementation in Laying Hens from Two High-Performing Strains

**DOI:** 10.3390/ani15101392

**Published:** 2025-05-12

**Authors:** Ákos Szentgyörgyi, Vera Sommerfeld, Markus Rodehutscord, Korinna Huber

**Affiliations:** Institute of Animal Science, University of Hohenheim, 70599 Stuttgart, Germany; akos.szentgyoergyi@uni-hohenheim.de (Á.S.); v.sommerfeld@uni-hohenheim.de (V.S.); markus.rodehutscord@uni-hohenheim.de (M.R.)

**Keywords:** dietary *myo*-inositol, serotonin, metabolic inflammation, laying hen

## Abstract

The purpose of this study was to investigate the effects of dietary supplementation of *myo*-inositol (**MI**) on the blood metabolite profile in two high-performing laying hen strains. Diets were corn-soybean meal-based and not supplemented or supplemented with 1, 2, or 3 g MI/kg feed. Ten hens per strain (Lohmann Brown-classic (**LB**) and Lohmann LSL-classic (**LSL**)) and diet were euthanized at the age of 30 weeks following a 4-week feeding period in metabolic units. Plasma and liver samples were taken from each individual hen. The Absolute-IDQ^TM^ p180 kit (Biocrates, Innsbruck, Austria) was used for metabolite profiling in plasma, plasma insulin, and liver triglyceride content were determined by a chicken-specific ELISA and a spectrometric assay, respectively. Supply of dietary MI caused only minor changes in metabolite profiles despite increased plasma MI concentrations. These changes indicated weak anti-inflammatory and antioxidative effects of MI feeding in laying hens. The effect of strain on metabolite profiles was more pronounced, indicating a higher level of metabolic inflammation in LB hens. By K-means cluster analysis, two clusters (**KMC1**, **KMC2**) of hens were identified with enhanced serotonin concentrations in KMC2 hens irrespective of strain and feeding. Further research is needed to assess the biological role of these findings.

## Simple Summary

*Myo*-inositol has several positive effects on metabolism in humans and mammals. However, knowledge about the effects of myo-inositol in laying hens is limited. Thus, in the present study, we investigated changes in the metabolite profiles of two distinct laying hen strains under different concentrations of dietary myo-inositol supplementation. Different diets resulted in differences in myo-inositol, methionine sulfoxide, symmetric dimethylarginine, and kynurenine concentrations in plasma. Moreover, several metabolites were altered between the two strains, suggesting potential differences in metabolic pathways. These data indicate that myo-inositol influenced the metabolism of laying hens. Notably, we identified some hens, irrespective of strain and diet, that appeared to express a metabolic condition characterized by elevated concentrations of serotonin and other biogenic amines in plasma. These hens might have a less detrimental metabolic condition than the other hens; however, additional research is required to confirm these findings. These findings suggest that myo-inositol has minor effects on metabolism, but laying hens with special peripheral serotonin-related metabolism may be a potential target for future research.

## 1. Introduction

Inositol (cyclohexane-1,2,3,4,5,6-hexol) is a sugar cyclic polyalcohol, and its most abundant form is *myo*-inositol (**MI**). MI can be found in several organs in rats (brain, pituitary gland, muscle, liver, spleen, kidney, thyroid glands, and male reproductive tract) [1]. MI has different important roles in the body. It acts as an osmolyte in the brain and kidneys and is the structural basis for secondary messengers and plasma membrane components (inositol triphosphates and phosphatidylinositol phosphate lipids) [2,3]. Moreover, MI has an insulin-mimetic effect, which was observed in several human studies with insulin-resistant patients who suffered from polycystic ovary syndrome and were treated with oral MI supplementation [4]. In obese, insulin-resistant monkeys, dietary MI supplementation decreased postprandial glucose and insulin concentration in plasma [5]. The study by Donà et al. (2012) [4] also suggested that MI has antioxidative properties in humans suffering from polycystic ovary syndrome.

Recently, MI and its effects on metabolism and performance have also attracted attention in chicken research [6,7,8,9]. The MI concentration in plasma is affected by MI absorption in the intestine, endogenous MI synthesis, dephosphorylation of inositol phosphates, and the rate of MI degradation [10]. The catabolism of MI takes place in the kidney, and MI oxygenase is the key degrading enzyme, at least in mammals [11].

Studies in broiler chickens revealed contradictory results. Dietary inclusion of 3 and 30 g MI/kg feed did not influence growth performance [6], while successive replacement of dextrose with MI increased growth performance and improved feed efficiency [8]. Increasing MI availability by very high dosing of dietary phytase (6000 FTU), which degrades inositol hexakisphosphate (phytate) to MI, phosphate, and lower inositol-phosphates, resulted in enhanced concentration of free inositol in the gizzard and ileal digesta but not in the kidney and liver [9]. Stronger indications for metabolic effects of dietary MI arose in broilers supplemented with 3.8 and 3.5 g MI/kg feed in the starter and grower phases, respectively, which were associated with increased dopamine and serotonin concentrations in plasma [12]. Inclusion of 0.1% MI into the diet of 50-week-old Bovans Brown laying hens resulted in a suppression of feed intake, reduced egg production, and reduced deposition of eicosanoid fatty acids in egg yolk [13]. Furthermore, MI (0.1%) failed to provoke responses in hematological indices [14]. In Lohmann Brown-Classic (**LB**) and Lohmann LSL-Classic (**LSL**) laying hens, MI concentrations were determined in the digesta of the gastrointestinal tract, in eggs, and in plasma as affected by the progression of productive life span (week 10 to 60 of age) [7]. An interrelationship of MI-related pathways with systemic metabolic conditions was detected in LSL and LB laying hens, showing that a higher expression of the MI-degrading enzyme MI oxygenase in LB hens was associated with indicators of metabolic stress [14].

Thus, the present study aimed to evaluate the effect of a dietary MI supplementation in laying hens to assess the effects on plasma metabolite profiles. By using a targeted metabolomics approach, a plethora of pathways can be screened for effects, and potential pathways of MI effects can be identified to be examined in future research. This combination of MI feeding and metabolomics approaches is novel in laying hen research. Metabolite profiling is helpful to get insights into hens’ health and may result in biomarker identification, which helps to understand metabolic variations and also metabolic adaptations to feeding supplements such as MI. Hypothetically, since distinct variations in metabolism were already detected in LB and LSL hens [14], the response to dietary MI supplementation might also be different. Dietary MI supplementation in broilers indicated an effect mainly on serotonin and dopamine metabolism, which was hypothesized to be of advantage to the broiler’s metabolic health [12]. MI supplementation in laying hens might also ameliorate their egg performance, like the proven positive effects of MI in human fertility [15]. Besides metabolite profiling, insulin and liver triglyceride (**TG**) content were determined as indicators of a potentially disturbed glucose and lipid metabolism associated with laying performance.

## 2. Materials and Methods

This study was part of the interdisciplinary Research Unit P-Fowl: Inositol phosphates and *myo*-inositol in the domestic fowl: Exploring the interface of genetics, physiology, microbiome, and nutrition (https://p-fowl.uni-hohenheim.de (accessed on 18 March 2025)).

### 2.1. Animals and Housing

Animals and the experimental design of this trial were described in detail by Sommerfeld et al. (2025) [16]. Briefly, the experiment took place at the Agricultural Experimental Station of the University of Hohenheim and was approved by the Regierungspräsidium Tübingen, Germany (Project No. HOH67-21TE) in accordance with the German Animal Welfare Legislation. LB and LSL newly hatched female chicks were obtained from a breeding company (Lohmann Tierzucht GmbH, Cuxhaven, Germany), and standard growth practices were applied during the rearing period. At the age of 26 weeks, 80 randomly chosen hens (10 per strain and diet combination) were individually moved to metabolic units (Figures S1 and S2; size of unit 1 m × 1 m × 1 m; 1 chicken per unit) where they had a wire mesh floor, a nest box, a wooden perch, water cups, and a feeder fixed at the outside of these units. The photoperiod was 16 h of light and 8 h of darkness, and the temperature in the barn was set to 18–22 °C. Treatments were assigned in a randomized complete block design. Randomization was performed using Microsoft Excel 2019 (Microsoft Corporation, Redmond, WA, USA) random numbers.

### 2.2. Diets

A detailed description of the diets was provided in the companion paper by Sommerfeld et al. (2025) [16]. In brief, diets were based on corn and soybean meal. Four diets were created based on the varying MI supplementation, as follows: diet **MI0** without MI supplement, diet **MI1** supplemented with 1 g MI/kg feed, diet **MI2** supplemented with 2 g MI/kg feed, and diet **MI3** supplemented with 3 g MI/kg feed. MI was supplemented as a pure chemical (purity 99%) (Myo-Inositol +98%, A13586; Thermo Fisher GmbH, Kandel, Germany). The selected supplementation levels were intended to replicate either a partial or complete release of MI from feed-derived inositol hexakisphosphate (1 or 2 g MI/kg) or a therapeutic dosage comparable to that used in humans (3 g MI/kg). The experiment was designed as a 2 × 4 factorial arrangement of treatments (2 laying hen strains and 4 MI levels) with *n* = 10 per group. The experimental diets were provided to the hens after placing them individually in metabolic units at week 26 for a period of 4 weeks. Feed and drinking water were provided for ad libitum consumption throughout the experiment.

### 2.3. Plasma Sampling

At the age of 30 weeks, all birds were used for sampling. Before slaughtering, feed access was stopped for 1 h, followed by 1 h of ad libitum access to feed to standardize gut fill during subsequent slaughter. Each hen was anesthetized with a gas mixture of 35% CO_2_, 35% N_2_, and 30% O_2_ and euthanized via decapitation. Blood samples from the trunk were collected in tubes containing EDTA (for metabolite profiling and insulin measures) or sodium fluoride (for MI measures). After the collection, EDTA blood was centrifuged at 1000× *g* for 15 min at room temperature, while sodium fluoride blood was centrifuged at 2500× *g* for 10 min at 4 °C. Thereafter, EDTA plasma was aliquoted, and aliquots were shock-frozen in liquid nitrogen and kept on dry ice until storing at −80 °C, while sodium fluoride plasma was stored directly at −20 °C.

### 2.4. Tissue Sampling

After the blood sample collection, the hens were immediately eviscerated. A section of the right lobe of the liver (*Lobus hepatis dexter*) was obtained with surgical equipment to preserve the integrity of the tissue, washed in ice-cold physiological saline, cut into small pieces, shock frozen in liquid nitrogen, and collected in pre-chilled cryotubes. Tubes were kept on dry ice until stored at −80 °C.

### 2.5. Myo-Inositol Determination in Plasma

Measurement of plasma MI concentration was performed as described by Sommerfeld et al. (2018) [17]. In brief, plasma samples were derivatized in several steps and measured using a gas chromatograph-mass spectrometer (Agilent 5977A, Agilent Technologies, Santa Clara, CA, USA).

### 2.6. Insulin Determination in Plasma

Plasma insulin concentrations were measured using a commercial ELISA kit (Chicken Insulin ELISA Kit, MBS741090; MyBioSource, San Diego, CA, USA). This assay has high sensitivity (0.1 ng/mL) and specificity (according to the manufacturer, less than 5% cross-reactivity was observed with rat and mouse samples). Measurements were performed according to the manufacturer’s protocol using an Infinite^®^ 200 PRO microplate reader at 450 nm (Tecan Group Ltd., Männedorf, Switzerland). A standard curve was constructed (concentrations on the x-axis and optical density on the y-axis) by using the sigmoidal, four-parameter logistic curve model of Tecan Magellan™ software V7.2 (https://lifesciences.tecan.com/software-magellan (accessed on 9 January 2025)). The concentration was expressed in ng/mL; the inter-assay CV was 7.2%.

### 2.7. Triglyceride Determination in Liver

TG concentrations in the liver were measured using a commercial colorimetric kit (ab65336; Abcam, Cambridge, MA, USA). Measurements were performed according to the manufacturer’s instructions, except for some changes. Briefly, 100 mg of tissue samples were manually homogenized in 5% nonyl phenoxypolyethoxylethanol using a pestle. Samples for TG determination were slowly heated to 90 °C in a water bath for 5 min twice. After the second heating, samples were centrifuged for 2 min at 13,000× *g* using a microcentrifuge. Supernatants were diluted to 1:100 with distilled water. Diluted samples and standards (50 µL of each) were pipetted on a 96-well plate, and 2 µL cholesterol esterase/lipase was added into each well, mixed, and incubated for 20 min at RT. After the incubation, 50 µL TG Reaction mix was added to each well. The plate was mixed and incubated at RT for 60 min, protected from light. Finally, the absorbance was measured at 570 nm by using an Infinite^®^ 200 PRO microplate reader (Tecan Group Ltd., Männedorf, Switzerland). Concentrations were calculated with Tecan Magellan™ software V7.2 (https://lifesciences.tecan.com/software-magellan (accessed on 9 January 2025)). TG concentrations were given in nmol/mg protein.

### 2.8. Protein Quantification

Liver homogenates were diluted at 1:100 in 5% nonyl phenoxypolyethoxylethanol. Protein concentrations were determined by the method according to Bradford (Bradford Reagent, SERVA, Heidelberg, Germany) in triplicate.

### 2.9. Targeted Metabolomics Approach

Plasma samples were used for metabolite profiling using the Absolute-IDQ^TM^ p180 kit (performed by Biocrates, Innsbruck, Austria). Metabolites belonging to the following substrate classes: amino acids (**AA**), acylcarnitines (**AC**), biogenic amines, hexoses, lysophosphatidylcholines (**LPC**), phosphatidylcholines (**PC**), and sphingomyelins (**SM**) were measured. Plasma metabolite concentrations are provided in micromole per liter (µmol/L). The assay is based on phenyl isothiocyanate derivatization in the presence of internal standards followed by flow injection analysis-tandem mass spectrometry (FIA-MS/MS, for the analysis of AC, LPC, PC, SM, hexoses) and liquid chromatography-tandem mass spectrometry (LC–MS/MS, for the analysis of AA and biogenic amines) using a 4000 QTRAP^®^ (AB Sciex, Framingham, MA, USA) or a Xevo^®^ TQ-S micro (Waters, Milford, MA, USA) instrument with electrospray ionization source. The experimental metabolomics measurement technique is described in detail by patents EP1897014B1 and EP1875401B1. All measurements were performed according to the certified guidelines and protocols of Biocrates by applying validated analytical methods.

### 2.10. Bioinformatic and Statistical Analyses

Visualization of metabolite profiles and discriminant and cluster analyses were performed by using MetaboAnalyst 5.0 (https://www.metaboanalyst.ca (accessed on 8 November 2024)) [18]. Metabolite concentrations were normalized by logarithmic transformation, mean-centered, and divided by the square root of the standard deviation of each variable (Pareto scaling). Two data sets were created, one consisting of all data (**all data set**) (160 measurable metabolites of IDQ p180 panel; insulin, liver TG, and traits such as body weight (**BW**) at slaughter, number of eggs and average egg weight within 27 days, plasma, egg albumen and yolk MI concentration, and egg weight in oviduct at slaughter (the latter data are originally published by Sommerfeld et al. (2025) [16]) (Table S1). The second data set excluded the substrate classes of PC, LPC, and SM (**reduced data set**) (the remaining 58 metabolites belong to substrate classes of AA, biogenic amines, hexoses, and AC, and the above-mentioned other variables). Metabolites of interest (**MOI**) discriminating between dietary groups and hen strains were identified by Partial Least Squares Discriminant Analysis (**PLS-DA**) as a supervised method. The validity of PLS-DA predictive modeling was assessed by cross-validation and a permutation test. Subsequently, MOIs were identified by Variable Importance in Projection (**VIP**) scoring. Metabolites with VIP scores above 1.0 were selected for further analysis.

The observed high inter-individual variation in concentrations of MOI pointed to grouping factors beyond diet and strain. K-means clustering (**KMC**) is an iterative, centroid-based unsupervised learning algorithm used for data clustering. Applying this cluster analysis to the reduced data set, two divergent clusters (KMC1, KMC2) were detected. Significantly differentiating metabolites were assessed by volcano plot (*p*-value threshold = 0.05, fold-change threshold = 1.0).

The final statistical evaluation of all identified MOIs regarding influencing factors (diet, strain) and interactions was performed using the MIXED procedure and pairwise *t*-tests as post-hoc tests using the software package SAS (version 9.3; SAS Institute Inc., Cary, NC, USA). The individual hen was considered the experimental unit. The detailed description of the model can be found in Sommerfeld et al. (2025) [16]. The level of significance for all tests was set at *p* < 0.05. Values for the tables and figures were given as LSmeans +/− SEM. Figures were created using GraphPad Prism version 9.5.1 (GraphPad Software, La Jolla, CA, USA) and MetaboAnalyst 5.0 (https://www.metaboanalyst.ca (accessed on 8 November 2024)).

## 3. Results

### 3.1. Effects of Dietary Myo-Inositol Supplementation on Myo-Inositol and Insulin Concentrations in Plasma and Triglyceride Concentrations in Liver

**Figure 1:**
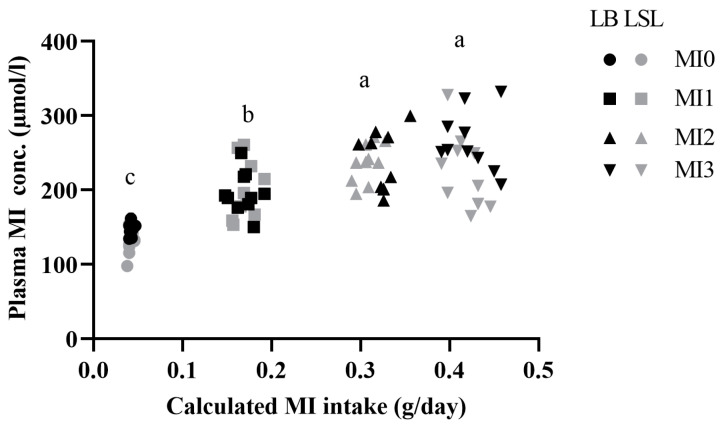
Association between calculated daily *myo*-inositol (**MI**) intake (g/day) and MI concentration in plasma (µmol/L) of Lohmann Brown-Classic (**LB**) and Lohmann LSL-Classic (**LSL**) laying hens at 4 different levels of MI supplementation. Each group contained 20 hens (10 per strain). MI0 contained 0 g supplemented MI/kg of feed, MI1 contained 1 g supplemented MI/kg of feed, MI2 contained 2 g supplemented MI/kg of feed, and MI3 contained 3 g supplemented MI/kg of feed. The comparisons of treatments were performed using the MIXED procedure using the software package SAS (*p* < 0.001). Different lowercase letters indicate significant differences among diet groups (*p* < 0.01).

Higher MI intake resulted in higher plasma MI concentration in LB and LSL hens, equally with increasing inter-individual variations at higher dosages of MI (Figure 1). The mean values were higher in the MI1, MI2, and MI3 groups compared to MI0. Furthermore, MI2 and MI3 had higher MI values than MI1, but MI2 and MI3 showed equal values. There was no significant difference between the strains and diets in terms of the insulin concentration in plasma and the TG concentration in the liver (Table S2).

### 3.2. Effects of Dietary MI Supplementation on Metabolite Profiles of Laying Hens

**Figure 2:**
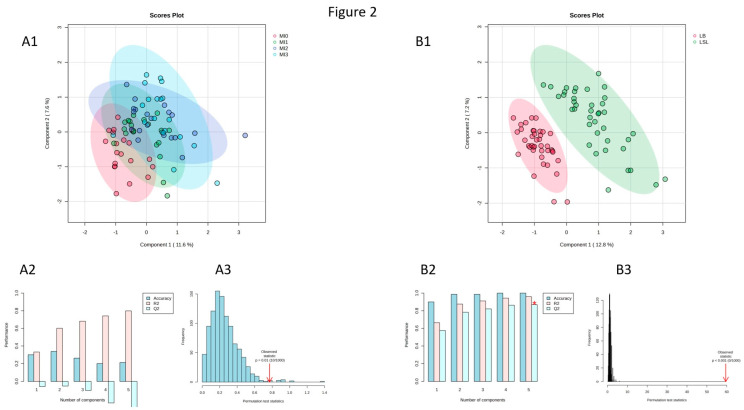
Partial Least-Squares Discriminant Analysis two-dimensional scores plots for the 4 diet groups, irrespective of strain (**A1**) and irrespective of diet (**B1**) (reduced data set without phosphatidylcholines, lysophosphatidylcholines, and sphingomyelins), including cross-validation (**A2**,**B2** (* represents maximum quality assessment statistic (Q2))) and permutation test (**A3**,**B3**). MI0 = no *myo*-inositol (**MI**) supplementation, MI1 = 1 g MI/kg feed, MI2 = 2 g MI/kg feed, MI3 = 3 g MI/kg feed; LSL = Lohmann LSL Classic; LB = Lohmann Brown-Classic.

PLS-DA analyses for the effects of diet and strain in the all data set revealed a lack of discrimination between the dietary groups (Figure S1A). However, a clear separation between the strains was observed (Figure S1B). Assessed by VIP scoring, most of the differentiating metabolites were members of the substrate classes PC, LPC, and SM. Since the interpretation of these findings is difficult due to a lack of knowledge about the meaning of these substrate classes in chickens, PLS-DA was performed using the reduced data set to discriminate between dietary groups and strains. A clear differentiation between dietary groups was again missing in the predictive model (Figure 2(A1)). Cross-validation (average Q2 = −0.206) (Figure 2(A2)) and permutation (*p* = 0.01) tests (Figure 2(A3)) confirmed that the discrimination between dietary groups was weak. However, the discrimination between strains was strong (Figure 2(B1)), as confirmed by cross-validation (average Q2 = 0.802) (Figure 2(B2)) and permutation (*p* < 0.001) tests (Figure 2(B3)).

### 3.3. Identification of Discriminating Metabolites Due to Myo-Inositol Supplementation

**Figure 3:**
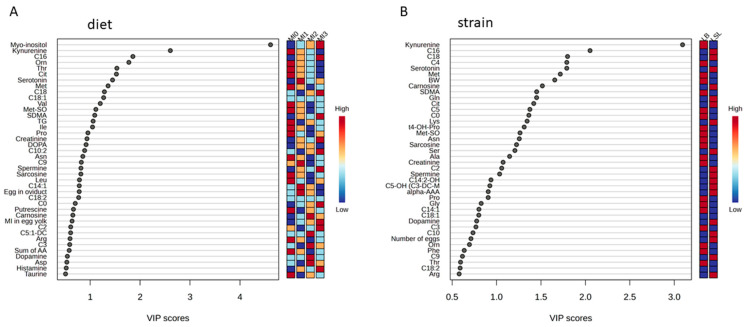
Variable importance in projection (**VIP**) scoring for the identification of metabolites differentiating groups according to diets (**A**) and strains (**B**). Reduced data set without phosphatidylcholines, lysophosphatidylcholines, and sphingomyelins. MI0 = no *myo*-inositol (**MI**) supplementation, MI1 = 1 g MI/kg feed, MI2 = 2 g MI/kg feed, MI3 = 3 g MI/kg feed; LSL = Lohmann LSL Classic; LB = Lohmann Brown-Classic.

To assess the metabolites that discriminate dietary groups, VIP scoring for the factor diet was performed. Forty metabolites or traits were identified (Figure 3A) and subsequently tested for significance by using a SAS mixed model. MI (Figure 1), ornithine (**Orn**), threonine (**Thr**), methionine sulfoxide (**Met-SO**), symmetric dimethylarginine (**SDMA**), kynurenine (**Kyn**), and Kyn/tryptophan ratio (**Kyn/Trp**) were significantly affected by diet (*p* < 0.05). For citrulline (**Cit**) concentrations, an interaction between diet and strain was observed (*p* < 0.05) (Table 1).

### 3.4. Identification of Discriminating Metabolites and Traits Due to Genetic Background

To assess the metabolites that discriminate the strains, VIP scoring for the factor strain was performed. Forty metabolites or traits were identified (Figure 3B) and subsequently tested for significance by using a SAS mixed model. Alanine (**Ala**), asparagine (**Asn**), glutamine (**Gln**), lysine (**Lys**), methionine (**Met**), serine (**Ser**), carnosine, creatinine, sarcosine, spermine, trans-4-hydroxyproline (**t4-OH-Pro**), carnitine (**C0**), acetylcarnitine (**C2**), butyrylcarnitine (**C4**), valerylcarnitine (**C5**), hexadecanoylcarnitine (**C16**) and BW were significantly different between the strains (Table S3). Furthermore, strain differences were found in the case of Cit, Met-SO, SDMA, Kyn, and Kyn/Trp ratio (Table 1).

### 3.5. Grouping of Laying Hens by Cluster Analysis

**Figure 4:**
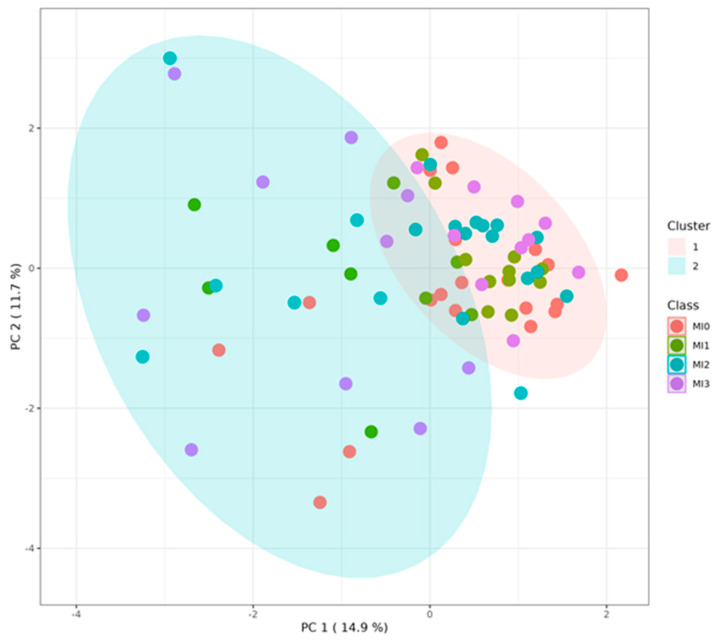
K-means cluster (**KMC**) analysis, including all hens of both strains and four dietary groups, resulted in two clusters (KMC1, KMC2). 1 = Cluster KMC1 (*n* = 59) included 32 = Lohmann Brown-Classic (**LB**) and 27 Lohmann LSL Classic (**LSL**) hens; from diets: MI0 16, MI1 15, MI2 14, MI3 14 hens. 2 = Cluster KMC2 (*n* = 21) included 8 LB and 13 LSL hens, from diets: MI0 4, MI1 5, MI2 6, MI3 6 hens. Reduced data set without phosphatidylcholines, lysophosphatidylcholines, and sphingomyelins. MI0 = no myo-inositol (**MI**) supplementation, MI1 = 1 g MI/kg feed, MI2 = 2 g MI/kg feed, MI3 = 3 g MI/kg feed.

**Figure 5:**
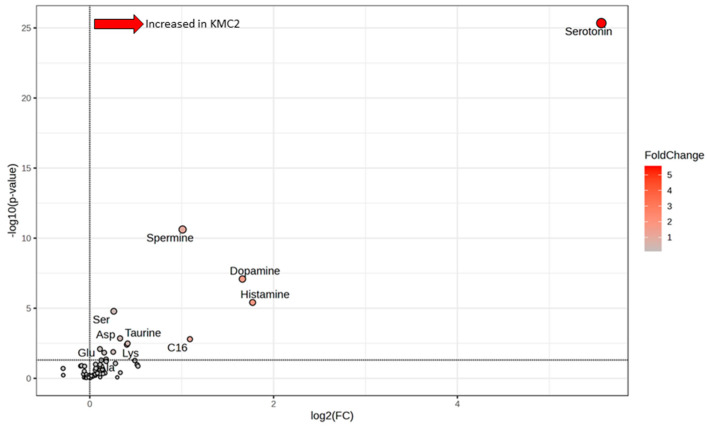
Volcano plot (threshold 1.0-fold changes, significance level *p* < 0.05) showing differences of metabolite concentrations in the plasma of laying hens of KMC1 (*n* = 59) and KMC2 (*n* = 21) clusters revealed by K-means cluster analysis. Reduced data set without phosphatidylcholines, lysophosphatidylcholines, and sphingomyelins. C16 = hexadecanoylcarnitine.

**Figure 6:**
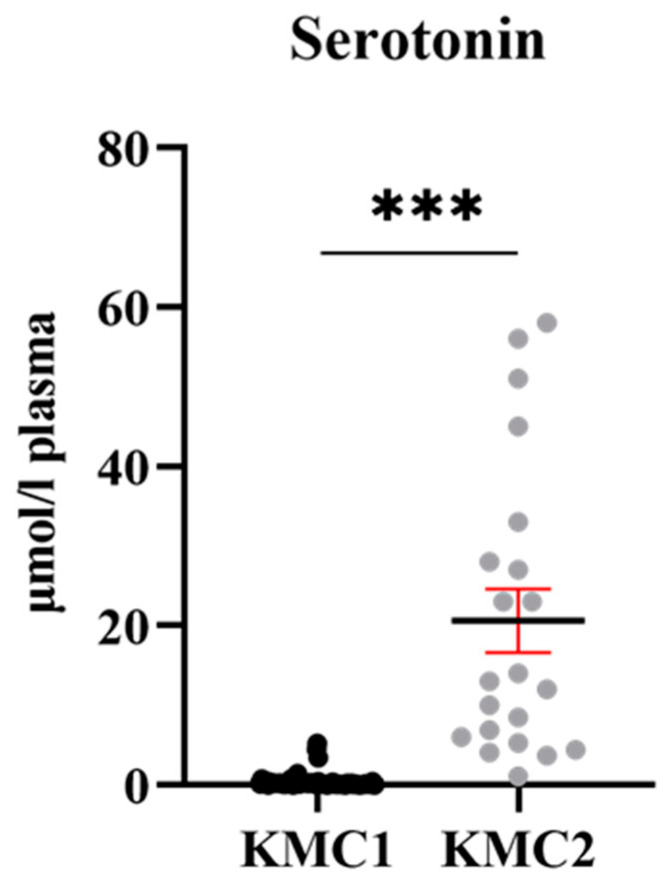
Concentration of serotonin in the plasma of laying hens belonging to cluster KMC1 (*n* = 21) and KMC2 (*n* = 59) was revealed by K-means cluster analysis. The comparisons of the 2 groups were performed using the MIXED procedure using SAS software. Significant difference was defined as *p* < 0.001 (***) between the clusters.

Two clusters were obtained by K-means clustering analysis, KMC1 and KMC2, irrespective of diets and strains. KMC1 consisted of 59 hens, whose metabolite profiles were very similar; KMC2 consisted of 21 hens, whose metabolite profiles very more variable (Figure 4). Analyzing the statistical differences between KMC1 and KMC2 by volcano plot revealed 13 metabolites highly (at least *p* ≤ 0.01) or slightly (at least *p* ≤ 0.05) significantly different, Ala, aspartate (**Asp**), glutamate (**Glu**), Lys, Ser, dopamine, histamine, serotonin, spermidine, spermine, taurine, C16 and sum of AA (Figure 5, Table 2). The most prominent difference between KMC1 and KMC2 was based on serotonin concentrations (Figure 6). While KMC1 had very low plasma serotonin concentrations in all hens of this cluster, KMC2 hens expressed a huge variation in their serotonin concentrations up to about 60 µmol/L. Plasma dopamine concentrations were only measurable in KMC2 cluster hens (Table 2).

## 4. Discussion

### 4.1. Myo-Inositol Supplementation and Metabolite Profiles in Laying Hens

Body MI content of chicken is most likely determined by dietary supplementation (free MI, phytate-bound MI) and by endogenous synthesis and breakdown, as in mammalian species [10]. MI might also have a positive impact on metabolic processes, membrane building, and signaling pathways such as insulin signaling, potentially leading to metabolic health and optimal performance of laying hens [10]. This study aimed to determine the effects of dietary supplementation with increasing amounts of MI on two high-performing laying hen strains. In another study, in pullets and hens without dietary MI supplementation, MI was correlated with many indicators of energy metabolism and immune functions, implicating better health in young hens with higher plasma MI concentrations irrespective of strain [14]. Based on these findings, dietary MI supplementation might promote better health in laying hens by increasing MI-related pathways and processes. MI had an anti-inflammatory and antioxidative effect when applied to human endothelial cells [19]. The calculated MI intake based on the chosen dosages increased the plasma MI concentrations (Figure 1). This confirms that dosages were high and long enough to provoke these changes. It is unclear if higher dosages or longer supplementation periods would have changed the plasma MI concentrations more pronouncedly. The changes in MI concentrations in plasma may reflect simultaneous changes in extracellular and intracellular spaces, causing adaptations in specific metabolic pathways. MI feeding-related metabolites detected in the present study are known to be indicators of low-grade systemic inflammation or metabolic inflammation, as discussed before. Cit supplementation appeared to reduce low-grade inflammation in the white adipose tissue of rats, which ameliorated systemic body health [20]. Furthermore, dietary L-Cit supplementation in broilers increased Cit, arginine (**Arg**), and Orn concentration in plasma and enhanced antioxidant and anti-inflammatory activity [21]. Thus, maintaining a higher concentration of Cit under MI1 and MI2 feeding in LSL hens might indicate that MI1- and MI2-fed LSL hens had a more anti-inflammatory status than MI1- and MI2-fed LB hens. MI effects on Cit could be based on strain-specific conditions, as indicated by a weak but significant interaction of both factors. Cit is an important precursor of AA, such as Arg [22]. Moreover, it is involved in nitric oxide production, and it may serve as an indicator of enterocyte function [23,24]. The connection between MI and Cit is unknown so far. However, in our study, LB hens expressed a more stable Cit concentration in plasma than LSL hens across all feeding groups; however, at MI3 feeding, the strain differences disappeared. For LSL, this MI3 Cit concentration was lower than in the non-supplemented group MI0. As a novel hypothesis, lower concentrations of MI may improve the antioxidative status and enterocyte function in LSL hens and LB hens. At higher dietary MI concentrations, decreased Cit indicated that LSL responded less sensitively to MI supplementation compared to LB hens. Further research is required to confirm these findings. The role of Orn, a non-proteinogenic AA, in laying hens is also unknown. However, in mice, L-Orn supplementation increased plasma insulin levels and improved general health conditions [25]. The insulin mimetic effect of MI could reduce the Orn need for insulin secretion induction, leading to decreased Orn concentration only in LSL hens. According to the present study, insulin concentrations in plasma were, however, not influenced by dietary MI supplementation. Thr, α-amino-β-hydroxybutyric acid, is an essential AA obtained from feed. It is well-known that Thr has an improving effect on protein synthesis, energy metabolism, and nutrient absorption in the gut [26]. With dietary MI supplementation, its plasma concentrations decreased in LB hens only, most likely due to increased use of this specific AA. This might indicate a slightly better metabolic performance and protein turnover promoted by Thr. Met-SO is an indicator of oxidative stress in physiological and pathological conditions and acts as a signal in transduction pathways; however, thresholds of Met-SO for differentiating conditions are not known for chicken [27]. Overall, dietary MI supplementation slightly decreased Met-SO in both strains, in general, at a lower level in LSL hens. SDMA concentration in human plasma was positively correlated with C-reactive protein, a marker for inflammation [28]. Its role in chickens has not been determined so far. SDMA concentrations in plasma rose slightly with increasing dietary MI supplementation in LSL hens only, but generally, SDMA was expressed at higher levels in LB hens. Kyn, an intermediate of Trp metabolism, is well described as an inflammatory marker in mammalian species [29]. One of the main enzymes of the Kyn pathway (indolamine 2,3-dioxygenase), which converts Trp to Kyn, is activated by pro-inflammatory cytokines. Thus, the Kyn/Trp ratio is also an indicator of inflammation in humans, and it positively correlates with pro-inflammatory cytokines [30,31]. Increasing dietary MI supplementation in LSL hens decreased the plasma Kyn concentrations. The Kyn/Trp ratio was reduced in LSL and LB hens with increasing MI supplementation, suggesting again an anti-inflammatory effect of MI. In general, LSL hens expressed lower values compared to LB hens. Taken together, dietary MI supplementation had only a weak effect on a few metabolites. However, most of them pointed to the same pathophysiological issue, the metabolic inflammation and its potential amelioration by MI in laying hens. It can further be hypothesized from the metabolite concentrations that LB hens (lower Cit, higher Met-SO, higher SDMA, higher Kyn, higher Kyn/Trp ratio) had a higher level of metabolic inflammation than LSL hens, irrespective of dietary MI supplementation.

### 4.2. Strain-Related Differences of Metabolite Profiles in Laying Hens

Besides the already mentioned metabolites, strains differed in further metabolites associated with substrate classes of biogenic amines, AA metabolism, and short-chain AC. LSL hens had strongly higher carnosine and slightly higher spermine concentrations in plasma. The dipeptide carnosine, β-alanyl-L-histidine, is well-known as an anti-aging peptide that expresses strong antioxidative features [32]. The polyamine spermine is endogenously synthesized from Orn and is involved in regulating cell growth, protein synthesis, apoptosis, and immune response [33]. Creatinine, sarcosine, and t4-OH-Pro concentrations in plasma were lower in LSL than LB hens. Creatinine indicates skeletal muscle mass; the lower body weight in LSL hens of the present study might be associated with lower muscle mass [34]. Sarcosine, produced in the liver and kidneys in mammalian species, was found to be enhanced in humans with abdominal obesity and metabolic syndrome [35]. Higher sarcosine concentrations in LB hens might be associated with more abdominal body fat and higher body weight. Metabolic syndrome is a complex disease, and one of the key symptoms is metabolic inflammation [36]. Thus, LB hens with a more pro-oxidative and pro-inflammatory condition likely suffered from metabolic inflammation. T4-OH-Pro is mostly found as a component of collagen. Thus, its concentration in plasma may depend on the collagen degradation rate [37]. Since pro-inflammatory cytokines increase the expression of matrix metalloproteinases, which belong to the most important enzymes in the process of collagen degradation [38], it can be hypothesized that higher t4-OH-Pro concentration in plasma indicates higher grade inflammation in laying hens. Furthermore, higher activity of matrix metalloproteinases could influence the stability of tissues and organs due to the weakening of structures belonging to the extracellular matrix. Thus, as a hypothesis, higher t4-OH-Pro concentrations in LB hens might be associated with structural dysfunctionality of tissues. LSL hens had significantly higher Gln, Lys, Ser, and lower Ala, Asn, Gly, Met, Phe, Pro, and Trp concentrations in plasma than LB hens. These differences in AA concentrations were not caused by differences in feed intake and were difficult to interpret, but supported the assumption that LB and LSL hens expressed strain-specific metabolic conditions [16]. C0, C4, and C5 concentrations were higher, while C2 and C16 concentrations were lower in LB compared to LSL hens. AC and C0 were indicators of mitochondrial activity due to their role in the fatty acid shuttle. Short-chain AC (C2, C4, C5) represent peroxisomal β-oxidation activity, and are also indicators of energy metabolism [39]. However, their exact physiological role is not fully clarified yet, especially in chickens.

### 4.3. Potential Meaning of Newly Identified Clusters of Laying Hens

By machine learning using K-means cluster analysis, 2 metabolite profile patterns were detected across strains and dietary MI supplementation levels. The KMC2 cluster had higher Ala, Asp, Glu, Lys, and Ser concentrations in plasma, and the sum of AA was higher than in the KMC1 cluster. Again, it is difficult to explain these findings in detail, but these differences were not based on differences in feed intake. An AA-related metabolite, taurine (2-aminoethanesulfonic acid), was also higher in KMC2 compared to KMC1. Taurine acts against oxidative stress and inflammation and was diminished in plasma in individuals suffering from cardiomyopathy; most likely, the hens of the KMC2 cluster might have had an improved antioxidative status [40]. C16 (palmitoylcarnitine) in plasma was also higher in KMC2, indicating well-performing mitochondria and effective fatty acid oxidation. Furthermore, palmitoylcarnitine was described as a surface-active molecule that can change membrane fluidity and thereby improve membrane functions [41]. The most prominent difference between KMC1 and KMC2 was based on 5 biogenic amines. KMC2 had higher concentrations of spermine, spermidine, histamine, dopamine, and serotonin. Especially, serotonin concentrations varied largely and ranged from 1.1 to 58 µmol/L in the plasma of KMC2 cluster hens (0.023 to 5.2 µmol/L in KMC1 cluster hens). The polyamines spermidine and spermine, synthesized endogenously by the host and by microbial metabolism, preserve mitochondrial functions, exhibit anti-inflammatory features, and slow down aging-related processes in mammals [42]. Serotonin (5-hydroxytryptamine) is mainly derived from duodenal enterochromaffin cells and is either secreted into the gut, modulating digestion, or secreted into the blood [43]. Most of the blood serotonin is clustered in thrombocytes and other blood cells [44]. Since this gut-derived serotonin cannot cross the blood-brain barrier, several peripheral effects of serotonin were investigated regarding inflammation and immune responses. It has been identified as a potent pulmonary vasoconstrictor in pulmonary hypertension syndrome in broilers, and it mediates oxidative stress and mitochondrial toxicity in steatohepatitis of mice [45,46]. However, the KMC clusters identified in the present study did not express differences in liver fat content. Furthermore, serotonin was enhanced in laying hens with footpad dermatitis [44]. Thus, laying hens of cluster KMC2 might suffer from an increased pro-inflammatory and pro-oxidative condition as indicated by high serotonin, irrespective of strain and diet. The higher biogenic amine and taurine concentration might reflect the metabolic response to serotonin-associated peripheral challenges. The underlying causes are unclear so far. A total of 26% of this study cohort expressed KMC2 metabolic condition, which might indicate a higher risk of suffering from production diseases such as fatty liver hemorrhagic syndrome. In future studies, besides characterization of metabolic condition, clinical symptoms need to be assessed to confirm that this metabolic condition is associated with production diseases. As a novel hypothesis, the peripheral serotonin-related metabolism appeared important for laying hens’ physiology and pathophysiology. Taken together, irrespective of strain and diet, some hens appeared to express a metabolic condition reflected by high serotonin and other biogenic amines, which most likely explained at least in part the inter-individual variation observed in both strains and feeding groups.

## 5. Conclusions

Dietary MI supplementation caused minor changes in the metabolic profiles. However, the affected metabolites may indicate that MI potentially decreased metabolic inflammation in laying hens. It can be hypothesized that LB hens had elevated metabolic inflammation compared to LSL hens, irrespective of dietary MI supplementation. Further characterization of the above-mentioned metabolic condition reflected by biogenic amines is needed to understand its biological meaning and to evaluate its potential impact on breeding approaches. Furthermore, these findings imply that MI supplementation could improve metabolic conditions. Future studies with a longer experimental period may show the potential relevance of these findings for laying hens’ health and performance.

## Figures and Tables

**Table 1:** Metabolites of interest as affected by diet and strain in the plasma of laying hens fed a basal diet or a basal diet plus 3 different levels of *myo*-inositol supplementation.

Metabolite (µmol/L)	Strain	MI0	MI1	MI2	MI3	TWA ^1^	*p*-Value
**Cit** ^2^	LB ^3^	10.86 ± 1.03 ^a,x^	8.97 ± 1.03 ^ab,y^	7.89 ± 1.03 ^b,y^	9.95 ± 1.03 ^ab,x^	Strain Diet Interaction	<0.01 0.098 0.013
	LSL ^4^	12.55 ± 1.03 ^a,x^	13.70 ± 1.03 ^a,x^	12.52 ± 1.03 ^a,x^	9.32 ± 1.03 ^b,x^		
				
**Orn** ^5^	LB	205.00 ± 15.71 ^a^	177.90 ± 15.71 ^a^	172.60 ± 15.71 ^a^	190.00 ± 15.71 ^a^	Strain Diet Interaction	0.107 0.017 0.292
	LSL	209.70 ± 15.71 ^a^	167.20 ± 15.71 ^b^	160.80 ± 15.71 ^b^	139.30 ± 15.71 ^b^		
				
**Thr** ^6^	LB	376.70 ± 25.41 ^a^	345.10 ± 25.41 ^ab^	298.10 ± 25.41 ^b^	304.10 ± 25.41 ^b^	Strain Diet Interaction	0.087 0.036 0.846
	LSL	327.40 ± 25.41 ^a^	297.10 ± 25.41 ^a^	280.30 ± 25.41 ^a^	284.00 ± 25.41 ^a^		
				
**Met-SO** ^7^	LB	13.20 ± 0.60 ^a,x^	11.46 ± 0.60 ^b^	11.81 ± 0.60 ^ab,x^	12.47 ± 0.60 ^ab,x^	Strain Diet Interaction	<0.001 0.035 0.092
	LSL	11.04 ± 0.60 ^a,y^	11.05 ± 0.60 ^a^	9.36 ± 0.60 ^b,y^	9.50 ± 0.60 ^b,y^		
				
**SDMA** ^8^	LB	0.44 ± 0.01 ^a,x^	0.43 ± 0.01 ^a,x^	0.42 ± 0.01 ^a,x^	0.45 ± 0.01 ^a,x^	Strain Diet Interaction	<0.001 0.044 0.052
	LSL	0.32 ± 0.01 ^b,y^	0.37 ± 0.01 ^a,y^	0.37 ± 0.01 ^a,y^	0.38 ± 0.01 ^a,y^		
				
**Kyn** ^9^	LB	0.30 ± 0.03 ^a,x^	0.33 ± 0.03 ^a,x^	0.24 ± 0.03 ^a,x^	0.24 ± 0.03 ^a,x^	Strain Diet Interaction	<0.001 0.039 0.700
	LSL	0.19 ± 0.03 ^a,y^	0.15 ± 0.03 ^ab,y^	0.13 ± 0.03 ^ab,y^	0.08 ± 0.03 ^b,y^		
				
**Kyn/Trp** **Ratio** ^10^	LB	3.43 ± 0.38 ^ab,x^	3.93 ± 0.38 ^a,x^	2.88 ± 0.38 ^b,y^	2.83 ± 0.38 ^b,x^	Strain Diet Interaction	<0.001 0.022 0.541
	LSL	2.22 ± 0.38 ^a,y^	1.81 ± 0.38 ^ab,y^	1.55 ± 0.38 ^ab,y^	0.92 ± 0.38 ^b,y^		
				

Data analysis was conducted with the software package SAS using the MIXED procedure and a pairwise *t*-test as a post-hoc test. Data are given as LSmeans ± SEM; *n* = 10 hens per diet group and strain. ^a,b^ Different superscript letters within a row indicate significant differences between diet groups (*p* < 0.05). ^x,y^ Different superscript letters within a column, and for each metabolite, indicate significant differences between strains (*p* < 0.05). *Myo*-inositol (**MI**) levels were supplemented as follows: MI0 = 0 g MI/kg feed, MI1 = 1 g MI/kg feed, MI2 = 2 g MI/kg feed, MI3 = 3 g MI/kg feed. In the case of kynurenine, missing values (*n* = 11), which were below the limit of detection, were replaced with 0.0001. ^1^ TWA = two-way ANOVA. ^2^ Cit = citrulline. ^3^ LB = Lohmann Brown-Classic. ^4^ LSL = Lohmann LSL-Classic. ^5^ Orn = ornithine. ^6^ Thr = threonine. ^7^ Met-SO = methionine sulfoxide. ^8^ SDMA = symmetric dimethylarginine. ^9^ Kyn = kynurenine. ^10^ Kyn/Trp ratio = kynurenine/tryptophan ratio.

**Table 2:** Metabolites of interest differentiating clusters in laying hens.

Metabolite (µmol/L)	KMC1 ^1^	KMC2 ^2^	*p*-Value
Ala ^3^	435.62 ± 15.74	483.68 ± 22.05	0.035
Asp ^4^	32.59 ± 1.33	40.81 ± 2.18	<0.01
Glu ^5^	210.11 ± 5.46	234.26 ± 8.82	0.019
Lys ^6^	238.27 ± 13.28	276.82 ± 16.68	0.011
Ser ^7^	749.89 ± 21.06	879.78 ± 26.99	<0.001
Dopamine ^8^	No data	0.29 ± 0.02	-
Histamine	0.03 ± 0.004	0.05 ± 0.004	<0.001
Serotonin	0.43 ± 1.22	20.62 ± 2.08	<0.001
Spermidine	0.32 ± 0.02	0.41 ± 0.03	0.010
Spermine	0.33 ± 0.03	0.64 ± 0.04	<0.001
Taurine	92.62 ± 7.75	124.64 ± 10.69	<0.01
C16 ^9^	0.01 ± 0.003	0.03 ± 0.004	<0.001
Sum of AA ^10^	5686.46 ± 110.35	6133.73 ± 155.79	<0.01

Data analysis was conducted with the software package SAS using the MIXED procedure. Data are given as LSmeans ± SEM, KMC1 *n* = 59, KMC2 *n* = 21. Metabolites were identified by volcano plot analysis (*p*-value threshold = 0.05; fold-change threshold = 1.0). In case of histamine 1 definitive (Q = 0.1%) outlier was excluded from the analysis. ^1^ KMC1 = cluster 1 derived from K-means cluster analysis. ^2^ KMC2 = cluster 2 derived from K-means cluster analysis. ^3^ Ala = alanine. ^4^ Asp = aspartic acid. ^5^ Glu = glutamic acid. ^6^ Lys = lysine. ^7^ Ser = serine. ^8^ Dopamine was not detectable (values were below the limit of detection) in KMC1. ^9^ C16 = hexadecanoylcarnitine. ^10^ Sum of AA = sum of amino acids.

## Data Availability

The datasets presented in this article are not readily available because the data are part of an ongoing study with a larger group of collaborators. Requests to access the datasets should be directed to korinna.huber@uni-hohenheim.de.

## Supplementary Materials

The following supporting information can be downloaded at: https://www.mdpi.com/article/10.3390/ani15101392/s1, Figure S1: Partial Least-Squares Discriminant Analysis 2D scores plots for the 4 diet groups irrespective of strain (A1) and irrespective of diet (B1) (all data set); Table S1: Egg-laying performance traits and myo-inositol concentrations in egg fractions of laying hens fed a basal diet or a basal diet plus 3 different levels of *myo*-inositol supplementation. Table S2: Insulin and triglyceride concentrations in plasma of laying hens fed a basal diet or a basal diet plus 3 different levels of *myo*-inositol (**MI**) supplementation; Table S3: Differential metabolite concentrations in plasma of laying hens fed a basal diet or a basal diet plus 3 different concentrations of *myo*-inositol (**MI**) supplementation. Figure S2. Empty metabolic units without feeders and waterers. Figure S3. Metabolic unit during the experiment.

## Abbreviations

The following abbreviations are used in this manuscript:

AA	Amino acid
AC	Acylcarnitine
Ala	Alanine
Arg	Arginine
Asn	Asparagine
Asp	Aspartate
BW	Body weight
Cit	Citrulline
C0	Carnitine
C2	Acetylcarnitine
C4	Butyrylcarnitine
C5	Valerylcarnitine
C16	Hexadecanoylcarnitine
Glu	Glutamate
Gln	Glutamine
KMC	K-means cluster
Kyn	Kynurenine
Kyn/Trp	Kynurenine/Tryptophan
LB	Lohmann Brown-Classic
LPC	lysophosphatidylcholine
LSL	Lohmann LSL-Classic
Lys	Lys
Met	Methionine
Met-SO	Methionine sulfoxide
MI	*Myo*-inositol
MOI	Metabolites of interest
Orn	Ornithine
PC	Phosphatidylcholines
PLS-DA	Partial Least-Squares Discriminant Analysis
SDMA	Symmetric dimethylarginine
Ser	Serine
SM	sphingomyelin
TG	Triglyceride
Thr	Threonine
t4-OH-Pro	Trans-4-hydroxyproline
VIP	Variable Importance in Projection
