# Supplementary material for: Metabolite Profiling Under Dietary Myo-Inositol Supplementation in Laying Hens from Two High-Performing Strains
Source: Animals (Basel). 2025 May 12;15(10):1392. doi: 10.3390/ani15101392 (PMC12108169; doi:10.3390/ani15101392)

SUPPLEMENTARY MATERIAL

Szentgyörgyi et al. 2025 Metabolite profiling under dietary myo-inositol supplementation in laying hens from two high-performing strains

Figure S1. Partial Least-Squares Discriminant Analysis 2D scores plots for the 4 diet groups irrespective of strain (A1) and irrespective of diet (B1) (all data set). Validity of models was assessed by cross validation (A2, B2) and permutation test (A3, B3). MI0 = no *myo*-inositol (MI) supplementation, MI1 = 1 g MI/kg feed, MI2 = 2 g MI/kg feed, MI3 = 3 g MI/kg feed; LSL = Lohmann LSL Classic; LB = Lohmann Brown-Classic.

A1

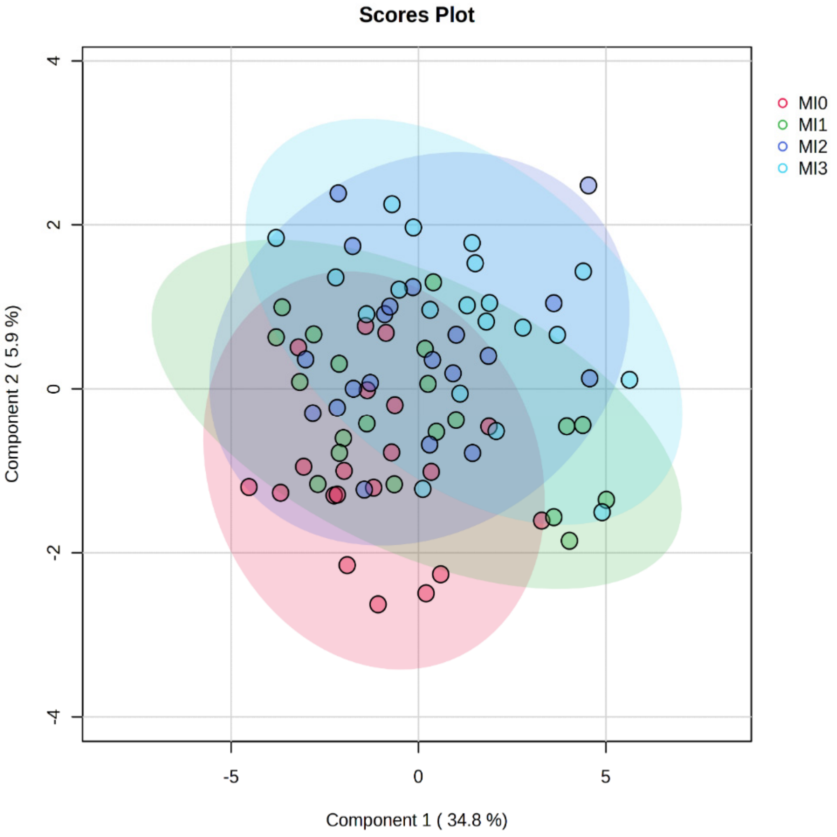

A2

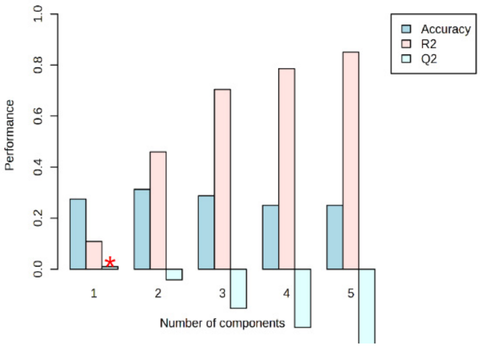

A3

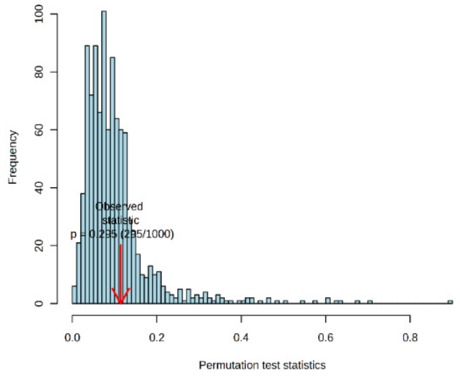

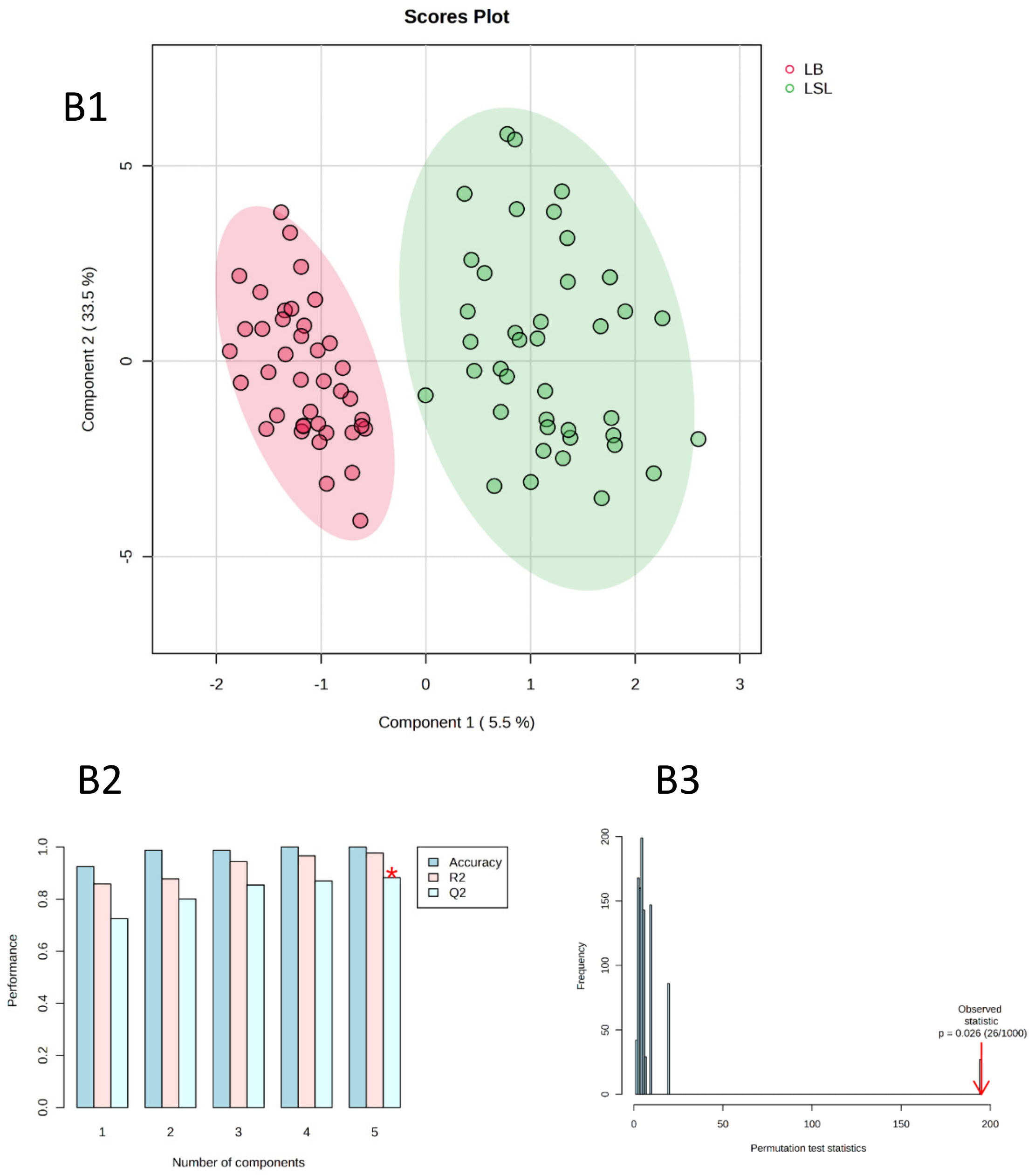

B1
Scores Plot
LB
LSL
Component 2 (33.5 %)
Component 1 (5.5 %)
B2
Accuracy
R2
Q2
Performance
Number of components
B3
Frequency
Observed statistic
p = 0.026 (26/1000)
Permutation test statistics

Table S1. Egg-laying performance traits and *myo*-inositol concentrations in egg fractions of laying hens fed a basal diet or a basal diet plus 3 different levels of *myo*-inositol supplementation.

Trait	Strain	MI0	MI1	MI2	MI3	TWA[1]	*P*-value
Number of eggs (g)[2]	LB[3]	25.80 ± 0.61	24.70 ± 0.61	24.60 ± 0.61	25.10 ± 0.61	Strain	$P < 0.01$
	LSL[4]	27.00 ± 0.61	26.50 ± 0.61	26.70 ± 0.61	26.70 ± 0.61	Diet	$P = 0.528$
						Interaction	$P = 0.896$
Average egg weight (g)[2]	LB	58.90 ± 0.95	59.20 ± 0.95	60.20 ± 0.95	61.50 ± 1.01	Strain	$P = 0.019$
	LSL	56.80 ± 0.95	58.70 ± 0.95	58.30 ± 0.95	57.90 ± 0.95	Diet	$P = 0.213$
						Interaction	$P = 0.405$
Egg albumen MI (µmol/g DM)	LB	3.91 ± 0.28	4.06 ± 0.28	4.50 ± 0.28	4.29 ± 0.29	Strain	$P = 0.910$
	LSL	3.98 ± 0.28	4.23 ± 0.28	4.16 ± 0.28	4.53 ± 0.28	Diet	$P = 0.103$
						Interaction	$P = 0.465$
Egg yolk MI (µmol/g DM)	LB	1.89 ± 0.06	2.01 ± 0.06	2.01 ± 0.06	2.19 ± 0.07	Strain	$P = 0.022$
	LSL	1.67 ± 0.06	1.81 ± 0.06	1.91 ± 0.06	2.07 ± 0.06	Diet	$P < 0.001$
						Interaction	$P = 0.573$

Data analysis was done with the software package SAS using the MIXED procedure and pairwise t-test as post-hoc test. Data are given as LSmeans ± SEM; n = 10 hens per diet group and strain.
Myo-inositol (**MI**) levels were supplemented as follows: MI0 = 0 g MI/kg feed, MI1 = 1 g MI/kg feed, MI2 = 2 g MI/kg feed, MI3 = 3 g MI/kg feed
Egg albumen MI and egg yolk MI results are already published by Sommerfeld et al. (2025) [16].
[1]TWA = Two-way ANOVA
[2]In 27 days
[3]LB = Lohmann Brown-Classic
[4]LSL = Lohmann LSL-Classic

Table S2. Insulin and triglyceride concentrations in plasma of laying hens fed a basal diet or a basal diet plus 3 different levels of *myo*-inositol supplementation.

Metabolite (µmol/l)	Strain	MI0	MI1	MI2	MI3	TWA[1]	*P*-value
Insulin	LB[2]	1.70 ± 0.32	1.46 ± 0.32	1.94 ± 0.32	2.31 ± 0.34	Strain	$P = 0.589$
	LSL[3]	1.76 ± 0.32	1.80 ± 0.34	1.67 ± 0.32	1.68 ± 0.32	Diet	$P = 0.719$
						Interaction	$P = 0.481$

Triglyceride	LB	5.74 ± 0.55	4.96 ± 0.55	5.80 ± 0.55	5.95 ± 0.55	Strain	*P* = 0.469
	LSL	6.52 ± 0.55	5.99 ± 0.55	5.92 ± 0.55	5.14 ± 0.58	Diet	*P* = 0.617
						Interaction	*P* = 0.360

Data analysis was done with the software package SAS using the MIXED procedure and pairwise t-test as post-hoc test. Data are given as LSmeans ± SEM; n = 10 hens per diet group and strain.
Myo-inositol (**MI**) levels were supplemented as follows: MI0 = 0 g MI/kg feed, MI1 = 1 g MI/kg feed, MI2 = 2 g MI/kg feed, MI3 = 3 g MI/kg feed.
[1]TWA = Two-way ANOVA
[2]LB = Lohmann Brown-Classic
[3]LSL = Lohmann LSL-Classic

Table S3. Differential metabolite concentrations in plasma of laying hens fed a basal diet or a basal diet plus 3 different concentrations of *myo*-inositol supplementation.

Metabolite (µmol/l)	Strain	MI0	MI1	MI2	MI3	P-value[1]
**Ala**	LB[2]	508.40 ± 28.90	464.20 ± 28.90	474.00 ± 28.90	499.90 ± 28.90	*P* < 0.01
	LSL[3]	426.60 ± 28.90	392.20 ± 28.90	403.70 ± 28.90	416.90 ± 28.90	
**Asn**	LB	203.80 ± 8.75	187.20 ± 8.75	184.50 ± 8.75	181.30 ± 8.75	*P* < 0.001
	LSL	165.20 ± 8.75	161.20 ± 8.75	150.70 ± 8.75	159.90 ± 8.75	
**Gln**	LB	835.10 ± 41.28	804.00 ± 41.28	799.90 ± 41.28	816.70 ± 41.28	*P* < 0.001
	LSL	980.70 ± 41.28	995.40 ± 41.28	969.30 ± 41.28	997.90 ± 41.28	
**Lys**	LB	235.60 ± 19.29	220.80 ± 19.29	209.20 ± 19.29	215.90 ± 19.29	*P* < 0.01
	LSL	295.10 ± 19.29	267.70 ± 19.29	248.60 ± 19.29	294.20 ± 19.29	
**Met**	LB	185.30 ± 10.95	156.10 ± 10.95	163.40 ± 10.95	160.60 ± 10.95	*P* < 0.001
	LSL	132.30 ± 10.95	139.70 ± 10.95	108.20 ± 10.95	109.20 ± 10.95	
**Ser**	LB	730.50 ± 37.55	728.60 ± 37.55	682.30 ± 37.55	745.10 ± 37.55	*P* < 0.01
	LSL	813.40 ± 37.55	857.30 ± 37.55	845.40 ± 37.55	869.30 ± 37.55	
**Carnosine**	LB	8.21 ± 0.58	7.87 ± 0.58	8.57 ± 0.58	8.83 ± 0.58	*P* < 0.001
	LSL	9.63 ± 0.58	11.08 ± 0.58	11.58 ± 0.58	10.13 ± 0.58	
**Creatinine**	LB	3.03 ± 0.15	3.07 ± 0.15	2.93 ± 0.15	3.19 ± 0.15	*P* = 0.017
	LSL	2.47 ± 0.16	2.79 ± 0.15	2.73 ± 0.15	2.66 ± 0.15	
**Sarcosine**	LB	9.94 ± 0.67	8.91 ± 0.67	8.52 ± 0.67	9.47 ± 0.67	*P* = 0.011

	LSL	7.98 ± 0.67	7.69 ± 0.67	7.11 ± 0.67	7.25 ± 0.67	
**Spermine**	LB	0.35 ± 0.07	0.34 ± 0.07	0.32 ± 0.07	0.37 ± 0.07	$P = 0.047$
	LSL	0.37 ± 0.07	0.52 ± 0.07	0.50 ± 0.07	0.50 ± 0.07	
**t4-OH-Pro**	LB	18.60 ± 0.81	17.80 ± 0.81	16.70 ± 0.81	18.50 ± 0.81	$P < 0.01$
	LSL	14.90 ± 0.81	14.30 ± 0.81	14.70 ± 0.81	16.10 ± 0.81	
**C0**	LB	12.74 ± 0.50	12.19 ± 0.50	13.10 ± 0.50	14.00 ± 0.50	$P < 0.001$
	LSL	10.55 ± 0.50	11.03 ± 0.50	10.99 ± 0.50	10.82 ± 0.50	
**C2**	LB	3.40 ± 0.19	3.08 ± 0.19	3.14 ± 0.19	3.39 ± 0.19	$P < 0.01$
	LSL	3.53 ± 0.19	3.79 ± 0.19	3.70 ± 0.19	4.05 ± 0.19	
**C4**	LB	0.11 ± 0.007	0.10 ± 0.007	0.09 ± 0.007	0.11 ± 0.007	$P < 0.001$
	LSL	0.08 ± 0.007	0.07 ± 0.007	0.08 ± 0.007	0.07 ± 0.007	
**C5**	LB	0.09 ± 0.006	0.09 ± 0.006	0.09 ± 0.006	0.09 ± 0.006	$P < 0.01$
	LSL	0.08 ± 0.006	0.07 ± 0.006	0.07 ± 0.006	0.07 ± 0.006	
**C16**	LB	0.01 ± 0.006	0.01 ± 0.006	0.01 ± 0.006	0.02 ± 0.006	$P = 0.010$
	LSL	0.02 ± 0.006	0.02 ± 0.006	0.03 ± 0.006	0.04 ± 0.006	
**BW**	LB	1934.60 ± 31.60	1915.60 ± 31.60	1941.10 ± 31.60	2000.90 ± 31.60	$P < 0.001$
**(g)**	LSL	1661.50 ± 31.60	1635.50 ± 31.60	1643.70 ± 31.60	1613.00 ± 31.60	

Data analysis was done with the software package SAS using the MIXED procedure and pairwise t-test as post-hoc test. Data are given as LSmeans ± SEM; n = 10 hens per diet group and strain.
Myo-inositol (**MI**) levels were supplemented as follows: MI0 = 0 g MI/kg feed, MI1 = 1 g MI/kg feed, MI2 = 2 g MI/kg feed, MI3 = 3 g MI/kg feed.
In case of 16 missing values, which were below the limit of detection, were replaced with 0.0001.
[1] P-values refer to the factor strain; factor diet and interactions were not significant
[2]LB = Lohmann Brown-Classic
[3]LSL = Lohmann LSL-Classic
Ala = alanine, Asn = asparagine, Gln = glutamine, Lys = lysine, Met = methionine, Ser = serine, t4-OH-Pro = trans-4-hydroxy-L-Proline, C0 = carnitine, C2 = acetylcarnitine, C4 = butyrylcarnitine, C5 = valerylcarnitine, C16 = hexadecanoylcarnitine, BW = body weight

Figure S2. Empty metabolic units without feeders and waterers.
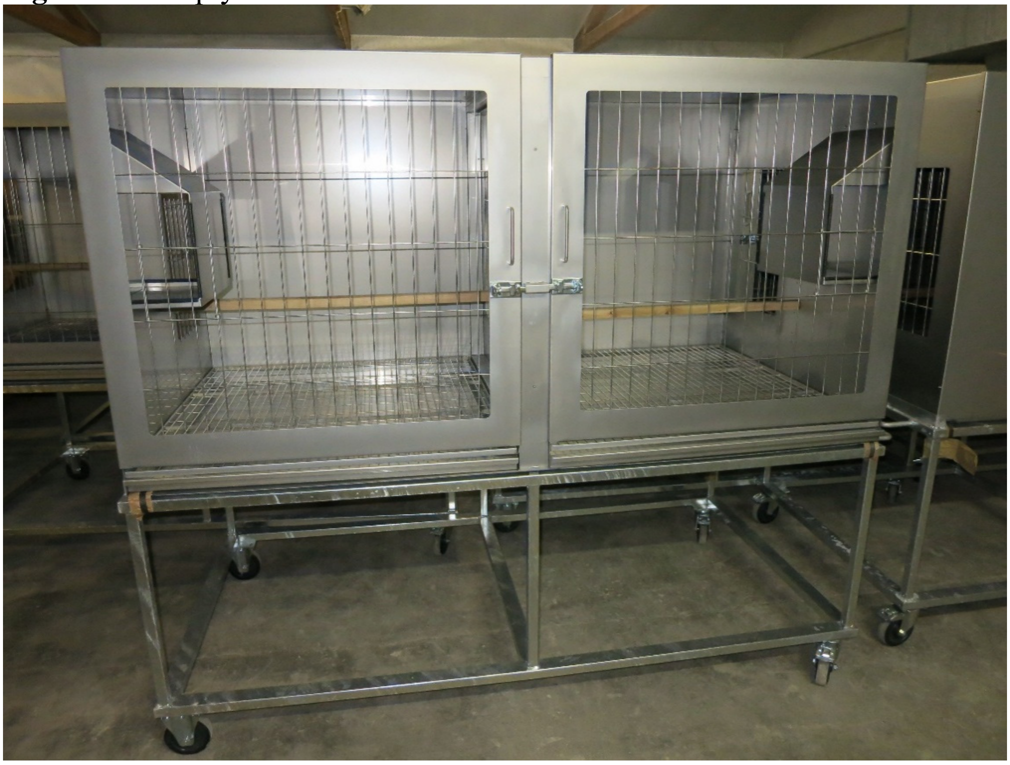

Figure S3. Metabolic unit during the experiment.

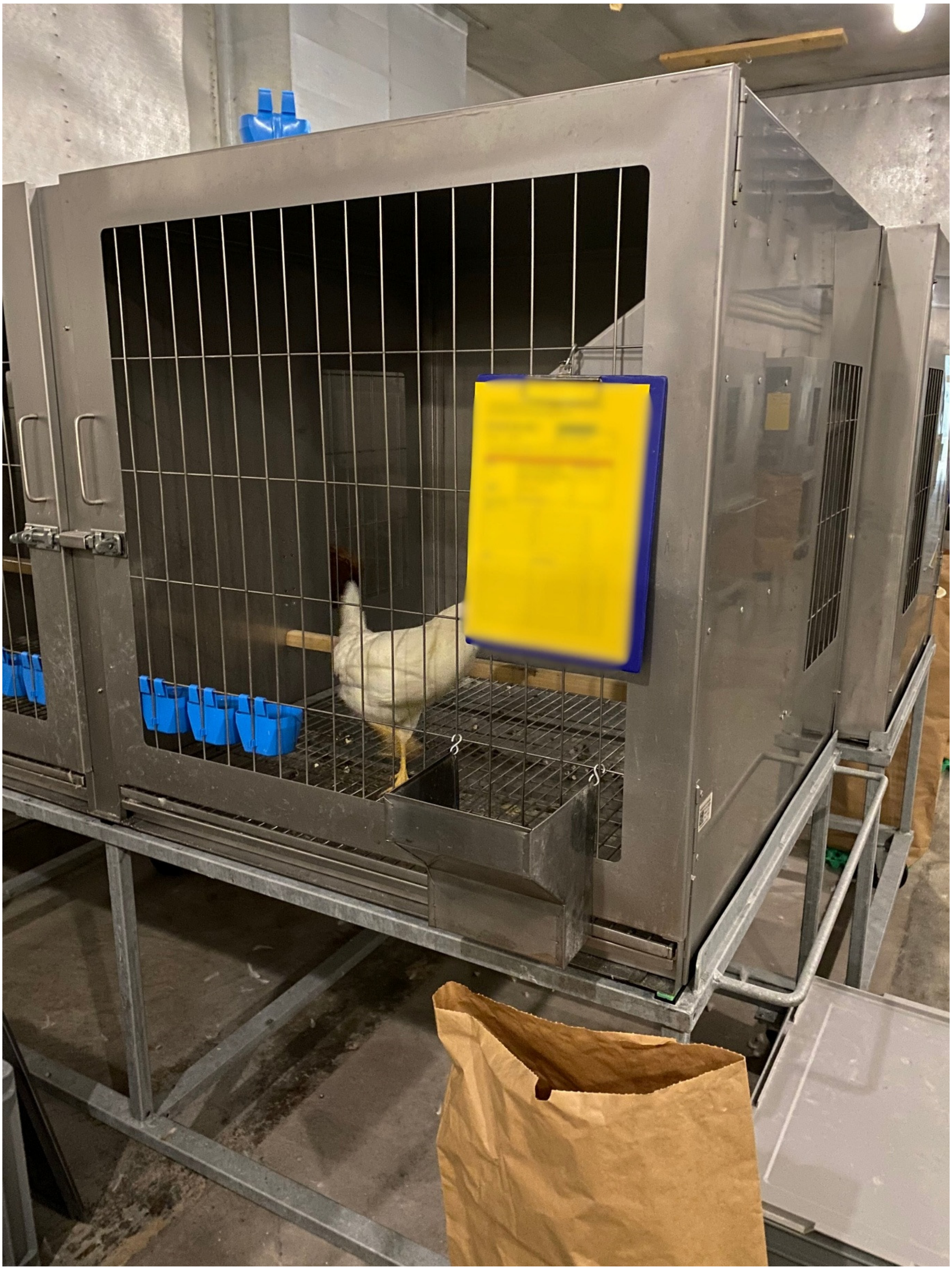

Supplement: Supplementary file 1 [file animals-15-01392-s001.zip › animals-3601813-supplementary.pdf]
